# Development of an optimized RNA-based murine norovirus reverse genetics system

**DOI:** 10.1016/j.jviromet.2010.07.006

**Published:** 2010-10

**Authors:** Muhammad Amir Yunus, Liliane Man Wah Chung, Yasmin Chaudhry, Dalan Bailey, Ian Goodfellow

**Affiliations:** Calicivirus Research Group, Section of Virology, Faculty of Medicine, Imperial College London, Norfolk Place, London W2 1PG, UK

**Keywords:** Reverse genetics, Norovirus, RAW 264.7

## Abstract

Murine norovirus (MNV), identified in 2003, is the only norovirus which replicates efficiently in tissue culture and as a result has been used extensively as a model for human noroviruses, a major cause of acute gastroenteritis. The current report describes the generation of a new approach to reverse genetics recovery of genetically defined MNV that relies on the transfection of *in vitro* transcribed capped RNA directly into cells. The use of the recently developed ScriptCap post-transcriptional enzymatic capping system, followed by optimized Neon mediated electroporation of the highly permissive RAW 264.7 cells, resulted in the rapid and robust recovery of infectious MNV. Transfection of cells capable of supporting virus replication but not permissive to virus infection, namely human or hamster kidney cells, also resulted in robust recovery of infectious virus without subsequent amplification by multiple rounds of re-infection. This latter system may provide a reproducible method to measure the specific infectivity of mutant norovirus RNA allowing the accurate quantitation of the effect of mutations on norovirus replication.

## 1 Introduction

Since the first demonstration that *in vitro* transcribed RNA from a cDNA clone of a positive strand RNA virus (Polio) was infectious in tissue culture (Racaniello and Baltimore, 1981), reverse genetics has proven to be an invaluable approach to studies on many aspects of virus replication and pathogenesis. In many cases, it has allowed an unprecedented ability to examine the effect of mutations not only on virus replication in tissue culture, but also on pathogenesis in the natural host. For some organisms this has led to the development of rationally attenuated viruses (Cavanagh et al., 2007) or improved vaccine candidates (Macadam et al., 2006).

**Fig. 1:**
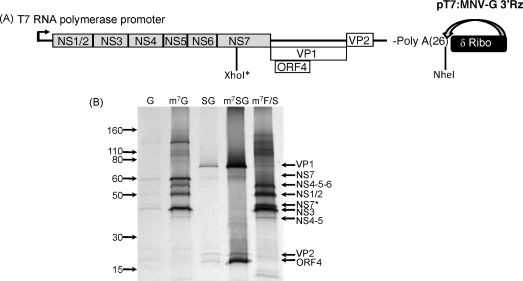
Murine norovirus full-length cDNA clone and translation of *in vitro* synthesised RNA. (A) Schematic of the infectious cDNA clone used in this study highlighting the positions of the four open reading frames, the T7 RNA polymerase promoter and the position of the 3′ ribozyme sequence. The asterisk highlights the position of the frame shift used to generate the mutated cDNA clone pT7:MNV-G 3′Rz F/S. (B) *In vitro* translation of *in vitro* transcribed murine norovirus genomic (G) or subgenomic RNA (SG). RNA was *in vitro* transcribed as described in Section 2.3 and in some cases enzymatically capped, highlighted by the prefix m^7^, prior to translation in rabbit reticulocyte lysates. Samples were resolved subsequently by 15% SDS-PAGE prior to exposure to a phosphoimager screen. Note that the protein assignments are based on the nomenclature proposed previously (Sosnovtsev et al., 2006). NS7* represents the truncated NS7 product generated as a result of the frame shift introduced in pT7:MNV-G 3′Rz F/S.

Murine norovirus (MNV), a member of the *Caliciviridae* family of small positive stranded RNA viruses, was first reported in 2003 (Karst et al., 2003) and still represents the only norovirus which replicates efficiently in tissue culture (Wobus et al., 2004). The discovery of MNV has allowed an unprecedented analysis of the role of viral sequences in norovirus translation, replication and pathogenesis in the natural host. As a result, MNV is used widely as a model system for the human noroviruses, a major cause of viral gastroenteritis in man (Wobus et al., 2006). The murine norovirus genome contains four reading frames (Fig. 1A); ORF1 encodes a large polyprotein which is cleaved co- and post-translationally to produce the viral non-structural proteins required for viral genome replication (NS1-7) (Sosnovtsev et al., 2006); ORF2 encodes the major capsid protein VP1; ORF3 encodes a minor structural protein whereas ORF4 encodes a protein of unknown function (Sosnovtsev et al., 2006).

A reverse genetics approach for MNV described previously relies on the transfection of a full-length cDNA construct of MNV into cells infected previously with fowlpox virus expressing T7 RNA polymerase (FPV-T7) (Chaudhry et al., 2007). This system was used subsequently to address many aspects of the norovirus life cycle including the identification of RNA structures important for norovirus replication (Simmonds et al., 2008), as well as mapping virulence determinants in the viral capsid protein (Bailey et al., 2008) and the 3′ untranslated region of the viral genome (Bailey et al., 2010). An RNA polymerase I promoter based reverse genetics system for the recovery of MNV has also been described (Ward et al., 2007), although published yields appear to be greater than 10-fold lower than those obtained using FPV-T7; 10^3^ plaque forming units/ml versus >5 × 10^4^ TCID50 per 35 mm dish for the Pol-I and FPV-T7 based systems respectively.

During the course of previous studies to generate a reverse genetics system (Chaudhry et al., 2007), the use of capped *in vitro* synthesised MNV RNA was examined as a possible reverse genetics approach as this has proven effective for other members of the *Caliciviridae*; both feline calicivirus (Sosnovtsev and Green, 1995) and porcine enteric calicivirus (Chang et al., 2005). However infectious virus was not recovered by transfection of capped *in vitro* transcribed MNV RNA into a number of efficiently transfected cell lines, including human 293T and hamster BHK cells, which although not permissive to infection by MNV viral particles, resulted in robust virus release when transfected with purified VPg-linked viral RNA (Chaudhry et al., 2007). In addition, transfection of *in vitro* transcribed capped viral RNA into the highly permissive, but more refractile to transfection RAW 264.7 murine macrophage cell line also failed to result in virus recovery. However, in this case, difficulties in the efficient delivery of RNA may have had a major impact on the ability to recover virus.

The current report describes the generation of an efficient and robust method for recovery of genetically defined murine norovirus from *in vitro* transcribed, post-transcriptionally capped RNA by transfection of several different cell types. This method displays >10-fold increase in virus yield when compared to the methods established previously and provides an additional approach with which to accurately quantify the effect of mutations within the norovirus genome on virus replication.

## 2 Materials and methods

### 2.1 Cells

Human embryonic kidney (293T) and murine macrophage cells (RAW 264.7) were obtained from ATCC and maintained in DMEM containing 10% foetal calf serum (FCS) at 37 °C with 10% CO_2_. BHK cells engineered to express T7 RNA polymerase (BSR-T7), as described previously (Buchholz et al., 1999), were obtained from Karl-Klaus Conzelmann (Ludwig Maximilians University, Munich, Germany) and maintained as described for 293 cells with the inclusion of 0.5 mg/ml G418. Note that BSR-T7 cells were used for this study simply due to their improved growth characteristics compared to BHK parental cells and the expression of T7 RNA polymerase in these cells has no effect on virus recovery as similar results are obtained using BHK (data not shown).

### 2.2 Plasmids and primers

pT7:MNV 3′Rz containing the wild type MNV-1 sequence under the control of a truncated T7 RNA polymerase promoter was described previously (Chaudhry et al., 2007). A modified version of this in which a frame shift was introduced into the NS7 region of the ORF1 open reading frame (pT7:MNV 3′Rz F/S) has also been described previously (Chaudhry et al., 2007). To generate a cDNA template for the synthesis of MNV subgenomic RNA, the PCR primers IGIC18 (TAATACGACTCACTATAGGGGTGAATGAGGATGAGTGATGGC) and 7400R (TTTTTTTTTTTTTTTTTTTTTTTTTTTTTTAAAATGCATCTAACTACCACAAAG) were used to amplify the viral subgenomic region, introducing a 5′ T7 RNA polymerase promoter and 30 nucleotides long 3′ poly-A tail.

### 2.3 *In vitro* transcription, RNA capping and RNA purification

Typically, transcription reactions contained 200 mM Hepes pH 7.5, 32 mM magnesium acetate, 40 mM DTT, 2 mM spermidine, 7.5 mM of each NTP, 25 ng/μl of linearised DNA template and 50 μg/ml of T7 RNA polymerase. Reactions were incubated at 37 °C for 2–7 h, treated with DNase at a final concentration of 0.1 unit/μl (New England Biolabs) then precipitated using lithium chloride, final concentration 2.5 M. RNA was resuspended in RNA storage solution (Ambion) and stored at −20 °C until required. The cDNA clones pT7:MNV 3′RZ or pT7:MNV F/S 3′RZ were linearised with NheI prior to *in vitro* transcription. RNA transcripts produced in this manner resulted in the inclusion of GCUAG at the 3′ end of the viral transcript due to the *NheI* overhang. RNA was capped using the ScriptCap system from Epicentre according to the manufacturer′s instructions. Briefly, up to 75 μg of RNA was denatured by heating to 65 °C for 10 min prior to rapid chilling on ice. The capping reaction was then set up by the addition of 10 μl of 10× capping buffer, 10 μl of 10 mM GTP, 0.5 μl of 20 mM S-adenosyl methionine, 2.5 μl of Scriptguard and 4 μl of ScriptCap enzyme mix in a total reaction volume of 100 μl. The reaction was incubated at 37 °C for 1 h then the RNA precipitated using lithium chloride. RNA was washed with 70% ethanol prior to resuspension in RNA storage solution (Ambion). RNA was stored at −20 °C until required.

Viral VPg-linked RNA was prepared as described previously (Chaudhry et al., 2007). Briefly, RAW 264.7 cells were infected with MNV using a multiplicity of infection of two TCID50 per cell, total RNA was then prepared from the infected cells between 12 and 18 h post-infection using the GenElute purification system (Sigma). The precise quantity of viral genome present in each preparation was not routinely determined, however analysis indicated that 1 μg of RNA prepared in this manner typically contained between 5 × 10^5^ and 5 × 10^6^ copies of MNV genomic RNA as determined using quantitative real-time RT-PCR (data not shown).

### 2.4 *In vitro* translation

*In vitro* translation reactions were performed using the Flexi rabbit reticulocyte lysate system from Promega. Reactions were prepared according to the manufacturer's instructions as described for capped mRNAs and programmed with 1 μg of RNA per 25 μl reaction. Reactions were incubated at 30 °C for 90 min prior to the addition of an equal volume of SDS-PAGE sample buffer. Samples were resolved subsequently on a 15% SDS-PAGE gel before exposure to phosphoimager screen.

### 2.5 Transfection of 293T and BSR-T7 cells

293T and BSR-T7 cells were transfected using Lipofectamine 2000 according to the manufacturer's instructions (Invitrogen). Briefly, 24 h prior to transfection, 7.5 × 10^5^ cells were seeded into a 35 mm dish in antibiotic free DMEM containing 10% FCS. Cells were then typically transfected with 1 μg of RNA, or amounts detailed in the figure legends, complexed with 4 μl of Lipofectamine 2000 for 20 min at room temperature in 200 μl of Optimem (Invitrogen). The transfection mixture was added to cells drop-wise prior to incubation at 37 °C. For virus recovery, cells were incubated for 24 h at 37 °C prior to freezing at −80 °C and subsequent titration by TCID50 on RAW 264.7 cells. Samples were prepared for western blot in an identical manner except rather than freezing at −80 °C, cells were lysed in RIPA buffer (50 mM Tris/HCl (pH 8.0), 150 mM NaCl, 1 mM EDTA, 1% Triton X-100, 0.1% SDS) and analysed subsequently by SDS-PAGE (15% acrylamide) and western blot using antisera to the viral polymerase NS7 or the minor capsid protein VP2.

### 2.6 Optimization of Neon mediated transfection of RAW 264.7 cells

In order to determine the optimum transfection conditions for the RAW 264.7 cell line highly permissive to MNV infection, cells were transfected with 1 μg of pGFPmax plasmid (Lonza) using the Neon transfection system. 6 × 10^6^ cells per transfection were harvested and washed once in PBS (Invitrogen). Cells were then resuspended in 100 μl of resuspension buffer and electroporated as detailed in Fig. 3A. Cells were immediately transferred to a 35 mm dish containing pre-warmed antibiotic free media and incubated at 37 °C for 24 h. Cells were first observed by bright field and fluorescence microscopy prior to harvesting and analysis by flow cytometry. Prior to flow cytometry analyses, cells were washed twice with ice-cold PBS and fixed with 4% paraformaldehyde for 15 min. After fixing, cells were rinsed with PBS and then analysed by flow cytometry using a CyAn™ ADP flow cytometer (Dako). Live cells were discriminated from dead cells and debris using forward and side scatter. The percentage of live cells expressing GFP was determined by comparison to mock transfected control cells.

## 3 Results

### 3.1 *In vitro* transcribed enzymatically capped MNV RNA translates efficiently *in vitro*

An infectious cDNA clone of MNV-1 in which the full-length viral cDNA is under control of a T7 RNA polymerase promoter, modified so that transcripts do not contain sequences of non-viral origin at the 5′ end of the T7 transcript has been described previously (Chaudhry et al., 2007). This plasmid, pT7:MNV 3′Rz also contains a unique *NheI* restriction site after a 27 nucleotide long poly-A tail, followed by a 3′ hepatitis delta virus ribozyme sequence (Fig. 1A). A derivative of this in which the NS7 RNA polymerase coding sequence contains a frame-shift (pT7:MNV 3′Rz F/S) has also been described previously (Chaudhry et al., 2007). Synthetic genomic RNA was prepared by T7 transcription of both plasmids as described in the materials and methods. Subgenomic RNA (sgRNA) was generated by transcription of a PCR product designed to introduce a T7 promoter at the 5′ end with a 30 nucleotide long 3′ poly-A tail (see Section 2 for details). Capped RNA was then generated using the ScriptCap enzymatic capping system and the effect of capping on the efficiency of the translation in rabbit reticulocyte lysates examined. Rabbit reticulocyte lysates were programmed with either capped or uncapped RNAs and protein synthesis levels examined (Fig. 1B). Whereas uncapped MNV genomic or subgenomic RNA failed to translate efficiently *in vitro*, enzymatically capped RNA typically produced >10-fold more protein *in vitro* (Fig. 1B).

### 3.2 *In vitro* transcribed enzymatically capped MNV RNA translates efficient and is infectious in cell culture

**Fig. 2:**
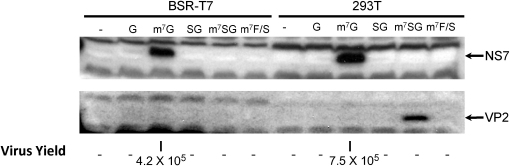
Recovery of murine norovirus using a heterologous cell system.Western blot analysis of either baby hamster kidney cells (BSR-T7) or human embryonic kidney cells (293T) transfected *in vitro* transcribed murine norovirus RNA. Cells were transfected with *in vitro* transcribed uncapped (G, SG) or enzymatically capped RNA (m^7^G, m^7^SG or m^7^F/S) as described in Section 2.3. *In* vitro transcribed genomic (G) or a derivative containing a frame shift in the NS7 region was prepared using the plasmids pT7:MNV-G 3′Rz and pT7:MNV-G 3′Rz F/S respectively. Subgenomic RNA was prepared using a PCR product engineered to contain a truncated T7 RNA polymerase at the 5′ end as described in Section 2.2. Cells were transfected with RNA and samples prepared for western blot using antisera to the viral NS7 and VP2 proteins 24 h post-transfection. Duplicate samples were also harvested and analysed subsequently for the presence of infectious virus by TCID50 on RAW 264.7 cells. Virus yield is show as TCID50 per transfection (1.5 × 10^6^ cells).

Previous work has highlighted that transfection of *in vitro* transcribed MNV RNA co-transcriptionally capped by the inclusion of cap analogue resulted in very low translation efficiency in cells (Chaudhry et al., 2007). As will be discussed later, this may be at least partly due to the inefficiency of capping and/or the stimulation of the interferon response as a result of transfecting 5′ phosphorylated RNA (Hornung et al., 2006; Nallagatla et al., 2007; Pichlmair et al., 2006). To examine if enzymatic capping of RNA could be used to overcome some of these issues and be efficiently translated in cells, a heterologous cell system, namely human embryonic kidney cells (293T) or baby hamster kidney cells (BSR-T7) were transfected with either capped or uncapped viral RNA transcripts then the levels of viral protein examined by western blot (Fig. 2). As expected, uncapped MNV genomic or subgenomic RNA did not produce detectable levels of viral proteins after transfection of either 293 or BSR-T7 cells. However, transfection of enzymatically capped RNA resulted in efficient and robust viral protein synthesis (Fig. 2). High levels of the viral NS7 protein, the product of open reading frame 1, was readily detected when capped viral genomic RNA was transfected into either cell type, whereas transfection of the viral subgenomic RNA lead to high levels of VP2 synthesis upon transfection of 293T cells only (Fig. 2). Note that VP2 expression was detected in BSR-T7 cells upon longer exposure but the expression levels were substantially reduced compared to the levels observed in 293T cells.

The presence of infectious virus in the cultures was also examined by subsequent titration on the highly permissive murine macrophage cell line RAW 264.7. High levels of infectious virus were readily detected in cultures transfected with capped viral genomic RNA only. Typical yields from a 35 mm dish (∼1.5 × 10^6^ cells) were >4 × 10^5^ TCID50, over 10-fold higher than the T7 RNA polymerase driven DNA based recovery system (Chaudhry et al., 2007) and >100-fold higher than a Pol-I based system (Ward et al., 2007). Previous analysis indicated that 293 and BSR-T7 cells, although competent for MNV replication as transfection of viral VPg-linked RNA results in high yields of infectious virus, are not permissive to infection, presumably due to the lack of a suitable virus receptor (Chaudhry et al., 2007). Therefore the yields of virus obtained after transfection of RNA into these cells represents a single cycle of replication only.

### 3.3 Optimization of RAW 264.7 cell transfection

**Fig. 3:**
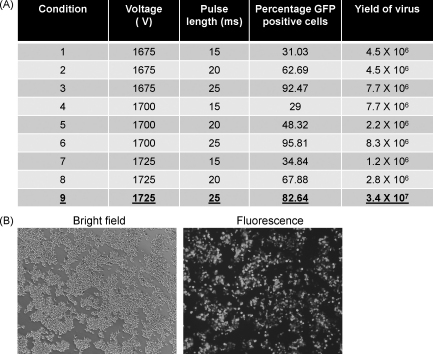
Neon mediated electroporation of RAW 264.7 cells. (A) Table depicting the results obtained by transfection of RAW 264.7 cells under various conditions with either 1 μg of GFP encoding DNA (pGFPmax) or 1 μg murine norovirus viral RNA (contained in a total RNA preparation isolated from infected cells). RAW 264.7 cells were transfected with 1 μg of plasmid using the conditions detailed in the table. The percentage of cells expressing GFP was determined at 48 h post-transfection using flow cytometry. Virus yield was determined as TCID50 per 6 × 10^6^ cells at 24 h post-transfection. (B) Bright field and fluorescence imaging of RAW 264.7 cells transfected with pGFPmax using condition 6 (1700 V, 25 ms) 48 h post-transfection.

Previous attempts to recover infectious MNV directly in the highly permissive RAW 264.7 murine macrophage cell line have been hindered by the inability to delivery viral nucleic acid efficiently to these cells (data not shown). To obtain high efficiency transfection of RAW 264.7 cells, the optimum conditions for the delivery of DNA were first examined by electroporation of pGFPmax using the Neon electroporation system (Invitrogen). A variety of conditions were examined and the percentage of GFP positive cells determined (Fig. 3A). Optimum conditions for DNA based delivery were determined to be 1700 V with a 25 ms pulse length. Under these conditions ∼95% of viable cells were GFP positive after transfection of 1 μg of DNA (Fig. 3B). To identify the optimal conditions for delivery of viral RNA, various transfection conditions were again examined however cells were transfected with 1 μg of MNV VPg-linked viral RNA, contained in a total RNA preparation from infected cells, and the virus yield determined 24 h post-transfection (Fig. 3A). Typical yields of infectious MNV after a single cycle of replication, namely 24 h post-transfection, were in excess of 1 × 10^6^ TCID50 per transfection (6 × 10^6^ cells). The optimal conditions for virus recovery were identified as 1725 V with a 25 ms pulse length and under these conditions typical yields were 3.4 × 10^7^ TCID50 at 24 h post-transfection of 1 μg of viral VPg-linked RNA (Fig. 3A).

### 3.4 Optimization of recombinant MNV recovery from RAW 264.7 cells

**Fig. 4:**
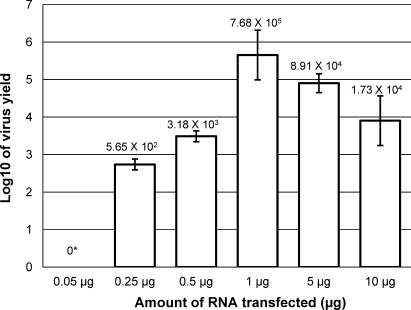
Optimization of murine norovirus recovery from RAW 264.7 cells. The virus yield obtained 24 h post-transfection of RAW 264.7 cells using varying amounts of *in vitro* transcribed post-transcriptionally capped MNV RNA. RAW 264.7 cells were transfected using the optimized conditions determined for virus recovery (1725 V, 25 ms). Virus yield is expressed as TCID50 per 6 × 10^6^ cells and represents the average of three repetitions. The asterisk highlights that no infectious virus was obtained but that the detection limit was ∼50 TCID50. Error bars represent the standard deviation.

To identify the optimum conditions which will allow the efficient and robust recovery of genetically defined noroviruses, including those with substantial growth defects, the optimum quantity of *in vitro* transcribed enzymatically capped RNA required for virus recovery was examined using the conditions demonstrated previously to give maximum yields from viral VPg-linked RNA. The yield of virus obtained by transfection of various quantities of RNA after a single cycle of replication, namely 24 h post-transfection, was examined (Fig. 4). These results indicate that maximum single cycle yields of virus were obtained after transfection of ∼1 μg of *in vitro* transcribed viral RNA, with typical yields of 7.7 × 10^5^ TCID50 per 6 × 10^6^ cells obtained (Fig. 4). At concentrations above 1 μg, increased cytopathic effect was observed (data not shown), but decreased yields of infectious virus were obtained.

### 3.5 Kinetic analysis of virus recovery from RAW 264.7 cells

**Fig. 5:**
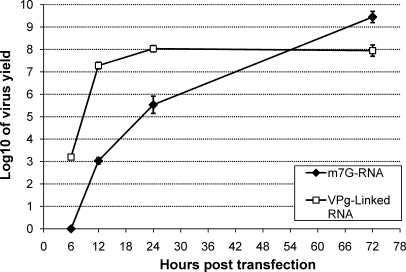
Time course analysis of murine norovirus recovery from RAW 264.7 cells. The virus yield from RAW 264.7 cells transfected with various amounts of either *in vitro* transcribed post-transcriptionally capped murine norovirus RNA or purified viral VPg-linked RNA. At various times post-transfection, cells were freeze–thawed to release infectious virus and the yield of virus determined by TCID50. Virus yield is expressed as TCID50 per 6 × 10^6^ cells and represents the average of three repetitions. Error bars represent the standard deviation.

The rate at which infectious virus could be recovered from transfected RAW 264.7 cells was examined in comparison to viral VPg-linked RNA contained in a total RNA preparation from infected cells (Fig. 5). Rapid recovery of infectious MNV was observed when RAW 264.7 cells were transfected with viral VPg-linked RNA, with >10^6^ TCID50 recovered as early as 12 h post-transfection and peak viral titres obtained at 48 h where >10^8^ TCID50 could be detected (Fig. 5). In contrast, transfection of *in vitro* transcribed post-transcriptionally capped MNV RNA resulted in slower recovery; >10^3^ TCID50 could be recovered by 12 h post-transfection but peak titres were obtained 72 h post-transfection. Maximal virus yields from RAW 264.7 cells transfected with *in vitro* transcribed RNA reached >10^9^ TCID50 per 6 × 10^6^ cells.

## 4 Discussion

Until recently, *in vitro* synthesised capped RNAs have been generated by co-transcriptional incorporation of cap analogues including m^7^G(5′)pppG (Yisraeli and Melton, 1989), into RNA. By the addition of an excess of cap analogue to *in vitro* transcription reactions, usually at a 10:1 or 4:1 ratio of cap analogue to GTP, much of the RNA produced contains a 5′ cap structure (m^7^G(5′)pppG(pN)). However, at best capping efficiencies of 75–80% can be achieved, with the remaining 20–25% of RNA containing a triphosphorylated 5′ end (Meis and Meis, 2007). Another drawback of this approach is that in many transcripts the cap analogue incorporates in the incorrect orientation (G(5′)pppm^7^G(pN)) and as such the RNA is translated inefficiently (Grudzien et al., 2004; Pasquinelli et al., 1995). Typically, in a transcription reaction in which a 4:1 ratio of cap analogue to GTP is used, approximately 80% of transcripts are capped, of which only 60% have incorporated the cap in the correct orientation. The use of anti-reverse cap analogues (ARCA) can overcome some of these limitations as due to the lack of one of the 3′ OH groups, incorporation can occur in the correct orientation only (Grudzien et al., 2004; Stepinski et al., 2001). However, it is impossible to obtain 100% capping efficiencies and therefore some RNA prepared in this manner remains uncapped possessing a triphosphorylated 5′ end.

Recent work on the characterisation of innate immune responses to virus infection have highlighted that 5′ phosphorylated RNA can stimulate the interferon system via the cellular RIG-I protein (Pichlmair et al., 2006). New studies have also indicated that in addition to a 5′ triphosphate, base-paired RNA sequences are required for interferon induction by RIG-I and PKR (Hornung et al., 2006; Nallagatla et al., 2007; Pichlmair et al., 2006). Hence, the use of capped *in vitro* synthesised viral RNA as a possible approach to reverse genetics is not only limited by the inability of some of the RNA to translate efficiently as a result of either the absence of a 5′ cap or an incorrectly incorporated 5′ cap, but also by the induction of interferon by those transcripts which are not capped. Given the reported sensitivity of murine norovirus to the interferon response (Changotra et al., 2009; Karst et al., 2003; Mumphrey et al., 2007), any induction of the interferon response is likely to result in a significant reduction in virus yield. Studies have also reported that the MNV genome possesses base-paired RNA structures at the 5′ end of the viral genomic RNA (Simmonds et al., 2008), hence, although not directly assayed in the current report, transfection of 5′ triphosphorylated MNV will inevitably result in interferon induction. Recent work has highlighted how type I and type II interferon can have a dramatic effect on MNV protein synthesis (Changotra et al., 2009).

The current report examines the use of enzymatically capped RNA, generated by the addition of an authentic cap structure to the 5′ end of *in vitro* transcribed RNA into by the tri-functional *Vaccinia* virus capping enzyme or guanyltransferase (Martin and Moss, 1975, 1976), commercially available from Epicentre as the ScriptCap capping system (Meis and Meis, 2007). The 5′ end of *in vitro* transcribed RNA is converted into a cap 0 structure post-transcriptionally, with efficiencies which approach 100% (Meis and Meis, 2007). RNA produced in this manner typically translate with a >5-fold increased efficiency in cells compared to RNA generated using traditional cap analogue or >3-fold greater efficiency than RNA produced using ARCA cap analogues (Meis and Meis, 2007). It is worth noting that by virtue of the mechanism of action of the enzymatic capping reaction, RNA produced in this manner will contain two 5′ G residues at the 5′ end of the viral genome rather than 1 i.e. m^7^GpppGpU. Whilst it is likely that this additional non-viral methylated G at the 5′ end may reduce the infectivity of the RNA, this may at least partially be compensated for by the lack of (or reduced) interferon induction achieved by transfection of RNA prepared in this manner.

Transfection of post-transcriptionally enzymatically capped RNA into heterologous cells, such as human 293T or hamster BSR-T7 kidney cells, allowed efficient recovery of virus in the absence of subsequent amplification due to re-infection. This approach may therefore be of use in quantifying the effect of specific mutations in viral proteins or RNA structures within the norovirus genome as virus yield would be the direct result of a single round of virus replication only. Transfection of synthetic capped genomic RNA resulted in yields of >4.5 × 10^5^ per 35 mm dish (1.5 × 10^6^ cells) (Fig. 2). Transfection of synthetic capped subgenomic RNA resulted in robust protein production in 293T cells only (Fig. 2), however upon longer exposure of the western blot displayed in Fig. 2, low levels of VP2 could also be detected in BSR-T7 cells (data not shown). Interestingly, co-transfection of viral genomic and varying amounts of subgenomic RNA did not increase virus yield in either cell type (data not shown).

By combining RNA capped using the *Vaccinia* virus capping enzyme with efficient transfection of the murine macrophage cell line RAW 264.7, it was possible to develop an efficient method of recovery of MNV in permissive cells. Optimized transfection conditions were determined using the Neon transfection system from Invitrogen (Fig. 3). In contrast to the majority of electroporation systems which use a cuvette based chamber, the Neon transfection system is based on the use of a pipette tip chamber which produces a more uniform electric field, resulting in higher cell viability (Kim et al., 2008). Using this system, transfection efficiencies of >95% were achieved and optimal condition for the recovery of MNV determined (Fig. 3A). Transfection of >1 μg of RNA resulted in substantial cytopathic effect at early times post-transfection, with reduced viral yield (Fig. 4). The observed increased cytopathic effect is most likely due the substantial over-expression of the viral proteins, prior to viral RNA replication, which resulted in premature cell death and reduced virus yield. In contrast, transfection of ∼1 μg of capped viral RNA resulted in >10^9^ TCID50 72 h post-transfection (Fig. 5), comparable to titres obtained following virus infection. Interestingly, as observed in 293T and BSR-T7 cells, co-transfection of the viral subgenomic RNA did not result in increased virus yield and in most cases produced substantially lower virus yields (data not shown).

In conclusion, a new method for norovirus reverse genetics was developed, which allows the rapid and robust recovery of genetically defined viruses in tissue culture. This method, when combined with mutational analyses, provides an additional tool with which to uncover the molecular mechanisms of norovirus translation, replication and ultimately virulence, aiding understanding of this group of significant pathogens.
